# HDAC6 Deacetylase Activity Is Critical for Lipopolysaccharide-Induced Activation of Macrophages

**DOI:** 10.1371/journal.pone.0110718

**Published:** 2014-10-16

**Authors:** Bing Yan, Songbo Xie, Zhu Liu, Jie Ran, Yuanyuan Li, Jian Wang, Yang Yang, Jun Zhou, Dengwen Li, Min Liu

**Affiliations:** 1 State Key Laboratory of Medicinal Chemical Biology, College of Life Sciences, Nankai University, Tianjin, China; 2 Key Laboratory of Immune Microenvironment and Disease of the Ministry of Education, School of Basic Medical Sciences, Tianjin Medical University, Tianjin, China; French National Centre for Scientific Research, France

## Abstract

Activated macrophages play an important role in both innate and adaptive immune responses, and aberrant activation of macrophages often leads to inflammatory and immune disorders. However, the molecular mechanisms of how macrophages are activated are not fully understood. In this study, we identify a novel role for histone deacetylse 6 (HDAC6) in lipopolysaccharide (LPS)-induced macrophage activation. Our data show that suppression of HDAC6 activity significantly restrains LPS-induced activation of macrophages and production of pro-inflammatory cytokines. Further study reveals that the regulation of macrophage activation by HDAC6 is independent of F-actin polymerization and filopodium formation; instead, it is mediated by the effects of HDAC6 on cell adhesion and microtubule acetylation. These data thus suggest that HDAC6 is an important regulator of LPS-induced macrophage activation and might be a potential target for the management of inflammatory disorders.

## Introduction

Macrophages with great diversity and plasticity are present in all tissues, where they are engaged in a range of activities including development, homeostasis, tissue remodeling, and immunity [1]. Primary macrophages are developed from monocytes which circulate in the blood and are recruited to the injured or infected tissues, where they undergo a series of alterations to become tissue resident macrophages [2]. Tissue resident macrophages are long-lived cells with modest proliferation, maintaining tissue homeostasis in the physiological conditions to phagocytose apoptotic cells and debris in the body [3]. In response to infection or injury, tissue macrophages are primed to the affected sites where they are activated to fulfill their role in innate and adaptive immune response [3], [4]. Activated macrophages produce proinflammatory cytokines/chemokines, which recruit other cells to the sites to eliminate the pathogens [5]. In addition, activated macrophages increasingly express the major histocompatibility complex class II antigens (MHC II), which are recognized by helper T cells through interaction with CD4 molecules and thereby leads to initiation of the adaptive immune response [6], [7]. However, deregulated activation of macrophages may also result in inflammatory diseases, such as sepsis, atherosclerosis, rheumatoid arthritis, and fibrosis [8], [9], suggesting that targeting macrophage activation would be an effective approach for the management of inflammatory disorders [10].

Activated macrophages are usually differentiated into classically activated macrophages (M1) or alternatively activated macrophages (M2) depending on different prototypical activating stimuli [11]. It is well characterized that bacterial lipopolysaccharide (LPS) and T helper type I (Th1) cytokines, such as interferon γ (IFN-γ), induce M1 phenotype, which produces pro-inflammatory cytokines to assist with the clearance of invading pathogens. By contrast, the stimulation with T helper type II (Th2) cytokines, such as interleukin 1β (IL-1β), IL-4, IL-13, or transforming growth factor β (TGF-β) results in the adoption of M2 phenotype, which produces anti-inflammatory cytokines to exert their function in tissue repair as well as protection from parasitic infections [12], [13]. Macrophages with different phenotypes are reversible in their specific microenvironments, frequently shifting their functional states to adapt for physiological or environmental changes [1], and the balance between M1 and M2 phenotypes is critical for the regulation of inflammatory disease progression [14].

A growing body of evidence reveals that macrophage activation towards these distinct phenotypes involves rearrangement of the actin and microtubule cytoskeleton components, which contribute to dramatic changes in cell shape to exert functionally distinct roles [14]. Histone deacetylase 6 (HDAC6), a unique member of the HDAC family, resides predominantly in the cytoplasm and regulates cytoskeletal dynamics through deacetylation of α-tubulin and cortactin [15], [16]. In addition, HDAC6 deacetylates many other important proteins, such as heat shock protein 90 (Hsp90) and Tat, as well as interacts with a number of cytoskeleton-associated proteins, such as end binding protein 1 (EB1), cytoplasmic linker protein 170 (CLIP-170), microtubule-associated protein 7 domain-containing protein 3 (Mdp3), and protein farnesyltransferase [17]–[22], suggesting an important role for HDAC6 in the regulation of cytoskeletal dynamics. Emerging evidence demonstrates that HDAC6 is associated with the secretion of pro-inflammatory cytokines, implicating a role in inflammatory disorders [23], [24]. However, whether HDAC6 is involved in macrophage activation remains elusive. In this study, our data show for the first time that suppression of HDAC6 restrains LPS-stimulated macrophage activation and that the regulation of macrophage activation by HDAC6 mainly results from its actions in microtubule acetylation.

## Materials and Methods

### Chemicals and antibodies

Tubacin, trichostatin A (TSA), sodium butyrate (NaB), 4′-6-diamidino-2-phenylindole (DAPI), anti-α-tubulin antibody, anti-acetylated α-tubulin antibody, and fluorescein isothiocyanate (FITC)- or tetramethylrhodamine (TRITC)-labeled phalloidin were purchased from Sigma-Aldrich. Anti-histone H4 antibody was purchased from Abcam. Anti-acetylated histone H4 antibody was purchased from Santa Cruz. Horseradish peroxidase-conjugated secondary antibodies and fibronectin were purchased from Millipore. FITC- or TRITC-conjugated secondary antibodies were obtained from JacksonImmuno Laboratories.

### Ethical declaration

Mouse experiments were carried out according to the guidelines of experimental animals and were approved by the Animal Care and Use Committee of Nankai University.

### Cell culture

RAW264.7 mouse macrophages were purchased from the American Type Culture Collection, cultured in the RPMI 1640 medium with 10% fetal bovine serum, and incubated with 5% CO_2_ at 37°C. For the isolation of bone marrow-derived macrophages (BMMs), 8- to 12-week-old C57BL6 mice were sacrificed by cervical dislocation, and the femurs and tibias were then separated. By flushing bones with cold phosphate-buffered saline (PBS), bone marrow cells were collected. Cells were then filtered through a 40-µm cell strainer (BD Biosciences). After washing with PBS, the cells were cultured in RPMI 1640 medium containing 10% FBS and 20 ng/ml M-CSF and incubated with 5% CO_2_ at 37°C.

### Cell transfection

Small interfering RNAs (siRNAs) targeting mouse HDAC6 (#1: 5′-GCACCAUGGUCAAGGAACA-3′; #2: 5′-CCAAUCUAGCGGAGGUAAA-3′; and #3: 5′-GGAGCUAAGCAGAGAAGCA-3′) and control luciferase (5′-CGTACGCGGAATACTTCGA-3′) were synthesized by RiboBio. siRNAs were transfected to cells by using lipofectamine 2000 reagent (Invitrogen) according to the manufacturer’s manuals.

### Quantitative RT-PCR

Total RNAs were isolated from RAW264.7 cells by using the TRIzol reagent (Invitrogen) and converted to cDNAs using the M-MLV reverse transcriptase (Promega). The amount of HDAC6 mRNA was measured by quantitative RT-PCR with the SYBR Green PCR master mix (primers: 5′-CATTGCTGCTTTCCTGCACATCCT-3′ and 5′-TCCAGGGACAGAATCAACTTGCCT-3′), and actin was used as an internal control (primers: 5′-CAGAAGGAGATTACTGCTCTGGCT-3′ and 5′-TACTCCTGCTTGCTGATC CACATC-3′).

### Immunoblotting

Immunoblot analysis of protein expression was performed as described previously [25], [26]. In brief, proteins were separated by sodium dodecyl sulfate-polyacrylamide gel electrophoresis and transferred onto polyvinylidene difluoride membranes (Millipore), followed by blocking with Tris-buffered saline containing 0.2% Tween 20 and 5% fat-free dry milk for 2 hours. Membranes were then incubated sequentially with primary antibodies and horseradish peroxidase-conjugated secondary antibodies. Specific proteins were detected with enhanced chemiluminescence detection reagent (Pierce) according to the manufacturer’s protocol.

### Cytokine production determination

RAW264.7 cells and primary BMMs were pretreated with DMSO or tubacin for 4 hours and then stimulated with LPS for 24 hours. The culture medium was collected and centrifuged at 2000 rpm for 5 minutes at 4°C. The supernatants were used for the determination of IL-6, IL-10, and TNF-α concentrations by using the alphaLISA kit (Perkin Elmer) according to the manufacturer’s protocol.

### Fluorescence microscopy

Cells were fixed with 4% paraformaldehyde in PBS for 20 minutes at room temperature, and then permeabilized with 0.05% Triton X-100 in PBS for 20 minutes. Cells were blocked with 2% BSA in PBS for 1 hour at room temperature. Cells were then sequentially incubated with primary antibodies and FITC- or TRITC-conjugated secondary antibodies. For F-actin staining, cells were incubated with FITC- or TRITC-conjugated phalloidin for 20 minutes. Nuclei were stained with DAPI for 5 minutes. Images were captured using a Zeiss fluorescence microscope or a Leica TCS SP5 confocal microscope as described previously [27], [28].

### Cell adhesion assay

Fibronectin or BSA was added to 96-well plates and incubated at 4°C overnight. Cells were labeled with calcein-AM at 37°C for 1 hour and then seeded onto the plates pre-coated with fibeonectin or BSA to allow for cell adhesion. One hour later, non-adherent cells were washed away with PBS. Images were taken with fluorescence microscopy before and after wash.

### F-actin sedimentation

Cells were collected and resuspended in F-actin stabilizing buffer (50 nM PIPES, 50 mM NaCl, 10 mM NaF, 5 mM MgCl_2_, 5% Glycerol, 0.1% NP-40, 0.1% Triton-X100, 0.02% β-mercaptoethanol, 1 mM ATP, and 2 mM PMSF). After homogenization, cell suspensions were centrifuged for 10 minutes to remove the nuclei and cell fragments. The supernatant was taken as the total actin control, and the remainder was centrifuged at 100,000 g for 1 hour. The supernatant was taken to detect G-actin, and the precipitate was dissolved and used for F-actin detection.

### Statistical analysis

All data were derived from three independent experiments, and presented as means ± SD. Student’s t-test and one-way analysis of variance (ANOVA) were performed for statistical analysis.

## Results

**Figure 1:**
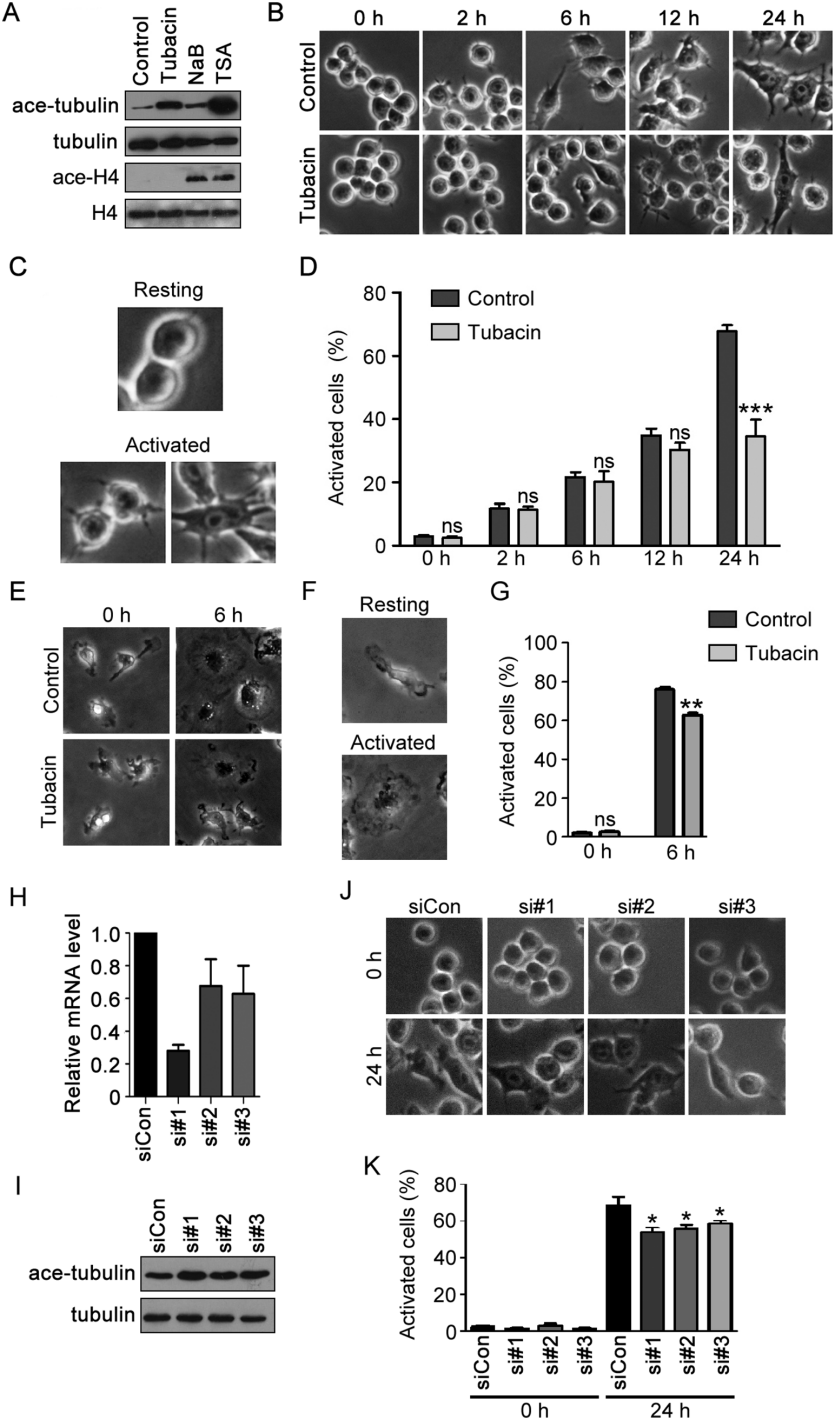
HDAC6 activity is important for LPS-induced macrophage activation. (A) RAW264.7 cells were treated with tubacin (1 µM), NaB (500 µM) or TSA (5 µM) for 4 hours. Cell lysates were then immunoblotted with antibodies against acetylated α-tubulin, α-tubulin, acetylated histone H4, and histone H4. (B) RAW264.7 cells were treated with tubacin (1 µM) for 4 hours and then stimulated with LPS (300 ng/ml) for 0, 2, 6, 12, and 24 hours. (C) Representative images of resting and activated macrophages. (D) Experiments were performed as in panel B, and the percentage of activated cells was analyzed. (E) Primary BMMs were treated with tubacin (1 µM) for 4 hours and then stimulated with LPS (300 ng/ml) for 0 and 6 hours. (F) Representative images of resting and activated BMMs. (G) Experiments were performed as in panel E, and the percentage of activated BMMs was analyzed. (H) Quantitative RT-PCR analysis of relative HDAC6 mRNA level in RAW264.7 cells transfected with control or mouse HDAC6 siRNAs for 36 hours. (I) Immunoblotting analysis of α-tubulin and acetylated α-tubulin in RAW264.7 cells transfected with control or mouse HDAC6 siRNAs for 72 hours. (J) RAW264.7 cells transfected with control or mouse HDAC6 siRNAs for 72 hours were stimulated with LPS (300 ng/ml) for 0 and 24 hours. (K) Experiments were performed as in panel J, and the percentage of activated cells was analyzed. ***, *p*<0.001; **, *p*<0.01; *, *p*<0.05; ns, not significant (*p*≥0.05).

LPS induces macrophage activation through the toll-like receptor 4 (TLR4) signaling and results in cell differentiation towards the M1 phenotype [29], a process involving cytoskeletal reorganization. To investigate the involvement of HDAC6 in LPS-induced macrophage activation, RAW264.7 cells were treated with tubacin, an HDAC6-specific inhibitor [30]. We found that the pan-HDAC inhibitor TSA increased the acetylation of both α-tubulin and histone H4, and the HDAC6-resistant HDAC inhibitor NaB increased histone H4 acetylation with minimal elevation of α-tubulin acetylation; by contrast, tubacin significantly increased α-tubulin acetylation without affecting histone H4 acetylation (Fig. 1A). These results demonstrate the specificity of tubacin in the suppression of HDAC6 activity.

Tubacin-treated macrophages were then stimulated with LPS, and the morphological changes of cells were examined 0, 2, 6, 12 and 24 hours later to verify macrophage activation (Fig. 1B). Activated macrophages are known to exhibit a spreading and elongated morphology with pseudopodium-like protrusions [31]. We defined the round shaped cells as resting cells and the elongated cells as activated cells (Fig. 1C). We found that the percentage of activated cells in the tubacin-treated group was markedly decreased compared with the control group after stimulation with LPS for 24 hours (Fig. 1D). To further investigate the role of HDAC6 in macrophage activation, we analyzed primary BMMs isolated from 8- to 12-week-old mice. Most of the BMMs showed a spreading morphology after treatment with LPS for 6 hours, and tubacin resulted in an impaired response of BMMs to LPS stimulation (Fig. 1E–1G).

To verify the function of HDAC6 in the regulation of macrophage activation, we knocked down its expression with specific siRNAs. Quantitative RT-PCR analysis of HDAC6 mRNA level revealed that these siRNAs effectively reduced HDAC6 expression (Fig. 1H), which was confirmed by the increase of tubulin acetylation following siRNA transfection (Fig. 1I). Importantly, knockdown of HDAC6 expression also decreased the activation of macrophages by LPS (Fig. 1J and 1K). Together, the above results indicate that HDAC6 plays an important role in LPS-induced macrophage activation.

**Figure 2:**
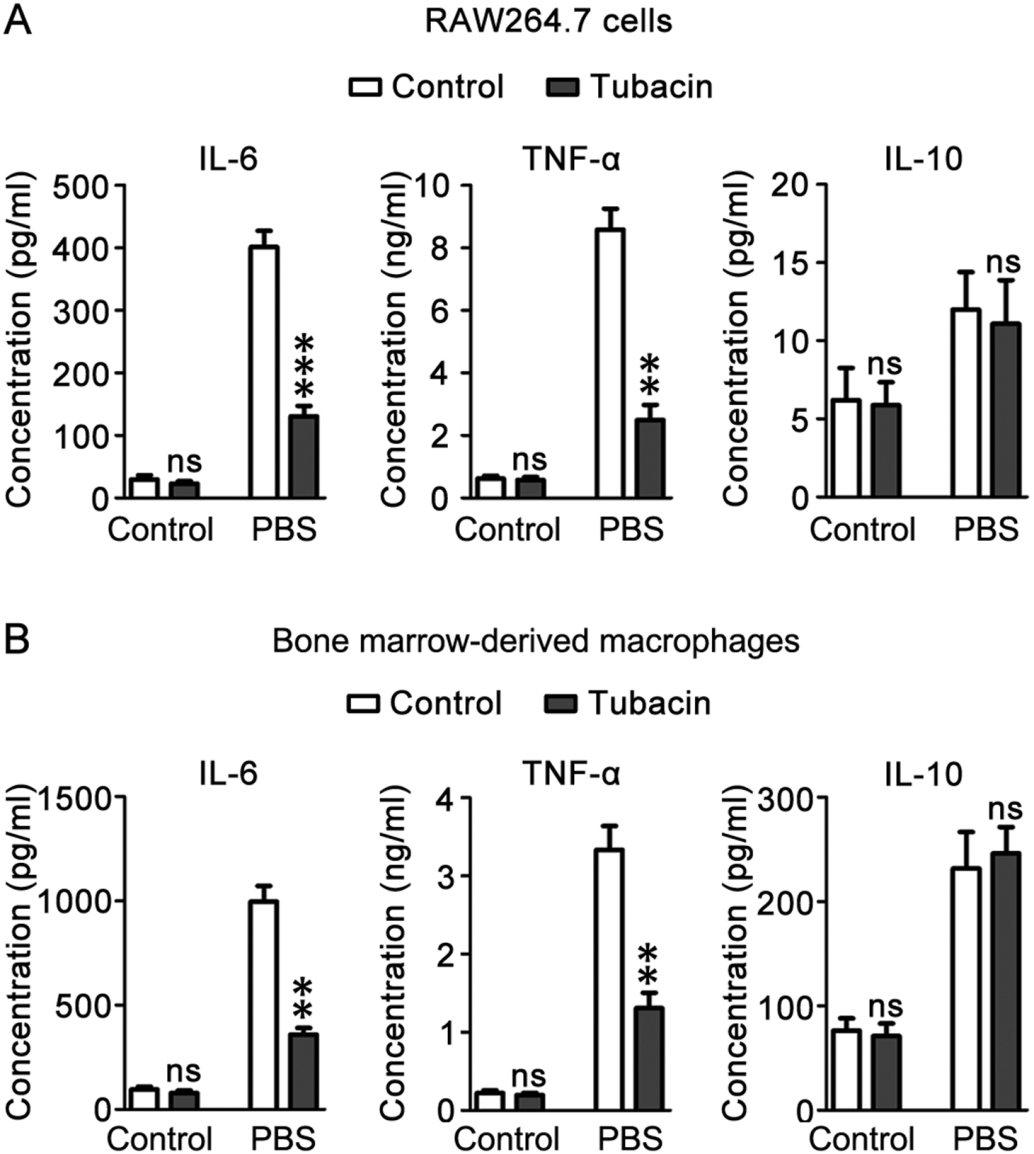
HDAC6 regulates LPS-induced M1 response. (A) RAW 264.7 cells pretreated with tubacin were stimulated with LPS (300 ng/ml) for 24 hours. The culture medium was then collected, and the concentrations of IL-6, TNF-α, and IL-10 were measured. (B) Primary BMMs pretreated with tubacin were stimulated with LPS (300 ng/ml) for 24 hours. The culture medium was then collected, and the concentrations of IL-6, TNF-α, and IL-10 were measured. ***, *p*<0.001; **, *p*<0.01; ns, not significant (*p*≥0.05).

Next we investigated the role of HDAC6 in LPS-induced M1 response. RAW264.7 cells and primary BMMs pretreated with tubacin were stimulated with LPS for 24 hours. The pro-inflammatory cytokines IL-6 and TNF-α (M1 markers) and the anti-inflammatory cytokine IL-10 (M2 marker) were then assessed. We found that LPS-induced secretion of IL-6 and TNF-α was dramatically decreased by tubacin in both RAW264.7 cells and primary BMMs; by contrast, LPS-induced IL-10 production was not obviously affected by tubacin (Fig. 2A and 2B). These results suggest that HDAC6 is critical for LPS-induced M1 response.

**Figure 3:**
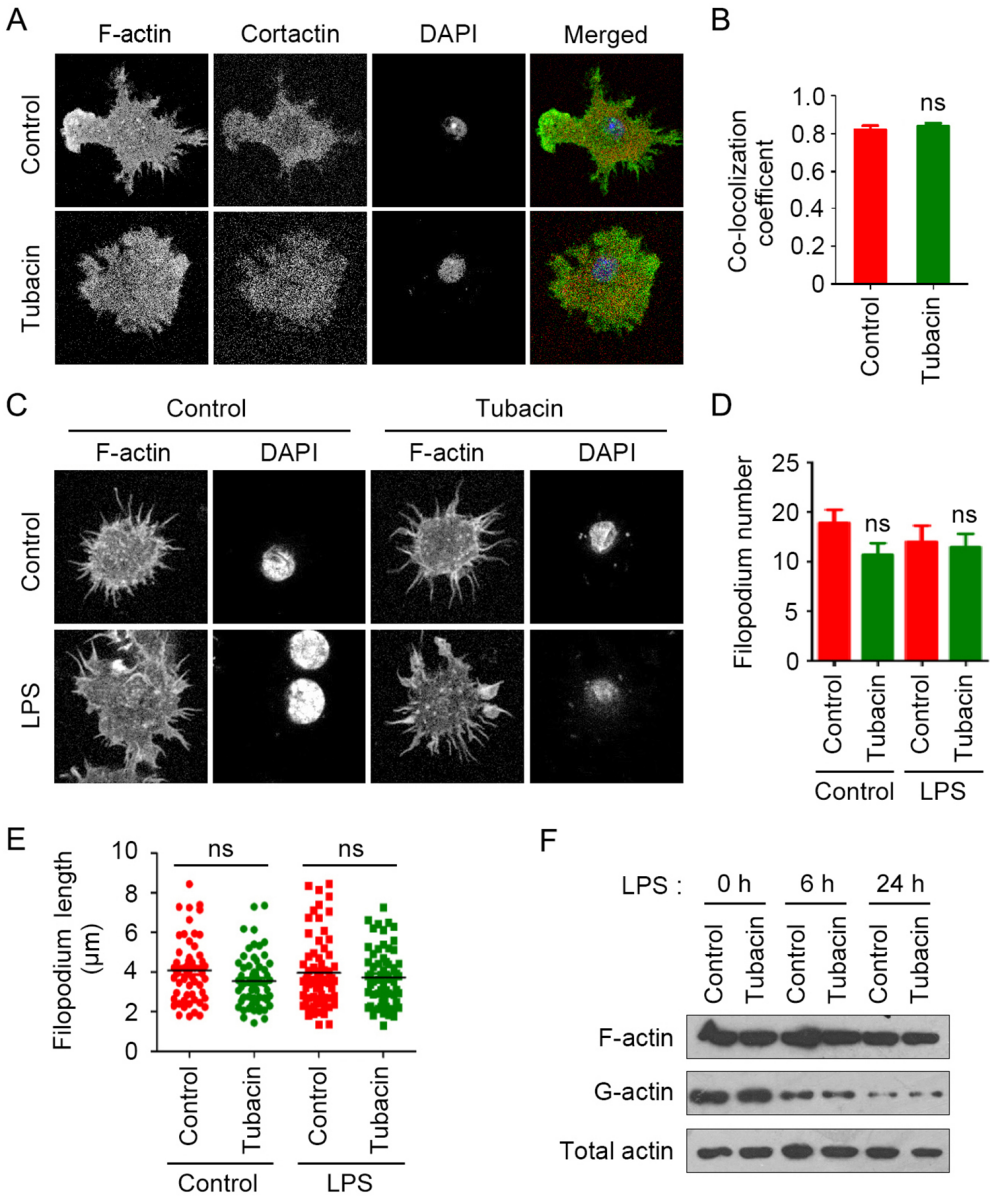
Effects of HDAC6 on actin polymerization and filopodium formation. (A) RAW264.7 cells were treated with tubacin (1 µM) for 4 hours and then exposed to LPS (300 ng/ml) for 24 hours. F-actin (green), cortactin (red), and nuclei (blue) were then stained, and images were captured with a laser scanning confocal microscope. (B) Experiments were performed as in panel A, and the Pearson’s correlation coefficient between F-actin and cortactin fluorescence pixels were calculated with ImageJ to measure their co-localization. (C) RAW264.7 cells were treated with tubacin for 4 hours and then with LPS for 6 hours. F-actin (green) and nuclei (blue) were then stained. (D, E) Experiments were performed as in panel C, and the filopodium number (D) and length (E) in each group were calculated. (F) RAW264.7 cells were treated with tubacin for 4 hours and stimulated with LPS for the indicated time. F-actin and G-actin were separated by ultracentrifugation and analyzed by immunoblotting. ns, not significant (*p*≥0.05).

Macrophage activation involves dramatic morphological changes of the cell shape due to the rearrangement of the actin and microtubule cytoskeleton. Cortactin, a key regulator of the actin cytoskeleton responsible for F-actin polymerization, is a substrate of HDAC6 deacetylase activity [16]. Thus, we investigated whether HDAC6 regulates macrophage activation through alteration of cortactin binding to F-actin. By immunostaining of F-actin and cortactin in LPS-stimulated macrophages, we found that inhibition of HDAC6 with tubacin did not significantly influence the co-localization of F-actin and cortactin (Fig. 3A and 3B).

Next we analyzed the role of HDAC6 in filopodium formation in LPS-stimulated macrophages by staining F-actin. As shown in Fig. 3C–3E, there was no significant difference in the number or length of filopodia between tubacin-treated group and the control group. To verify this finding, we examined F-actin polymerization by immunoblotting. By sedimentation of F-actin from the cellular homogenates, we found that there was no obvious change in the relative level of F-actin between control and tubacin-treated groups (Fig. 3F). Interestingly, although the relative amount of G-actin was reduced after the treatment of LPS, there was still no significant change between the tubacin-treated group and the control group (Fig. 3F). These results indicate that HDAC6 regulates macrophage activation independently of F-actin polymerization and filopodium formation.

**Figure 4:**
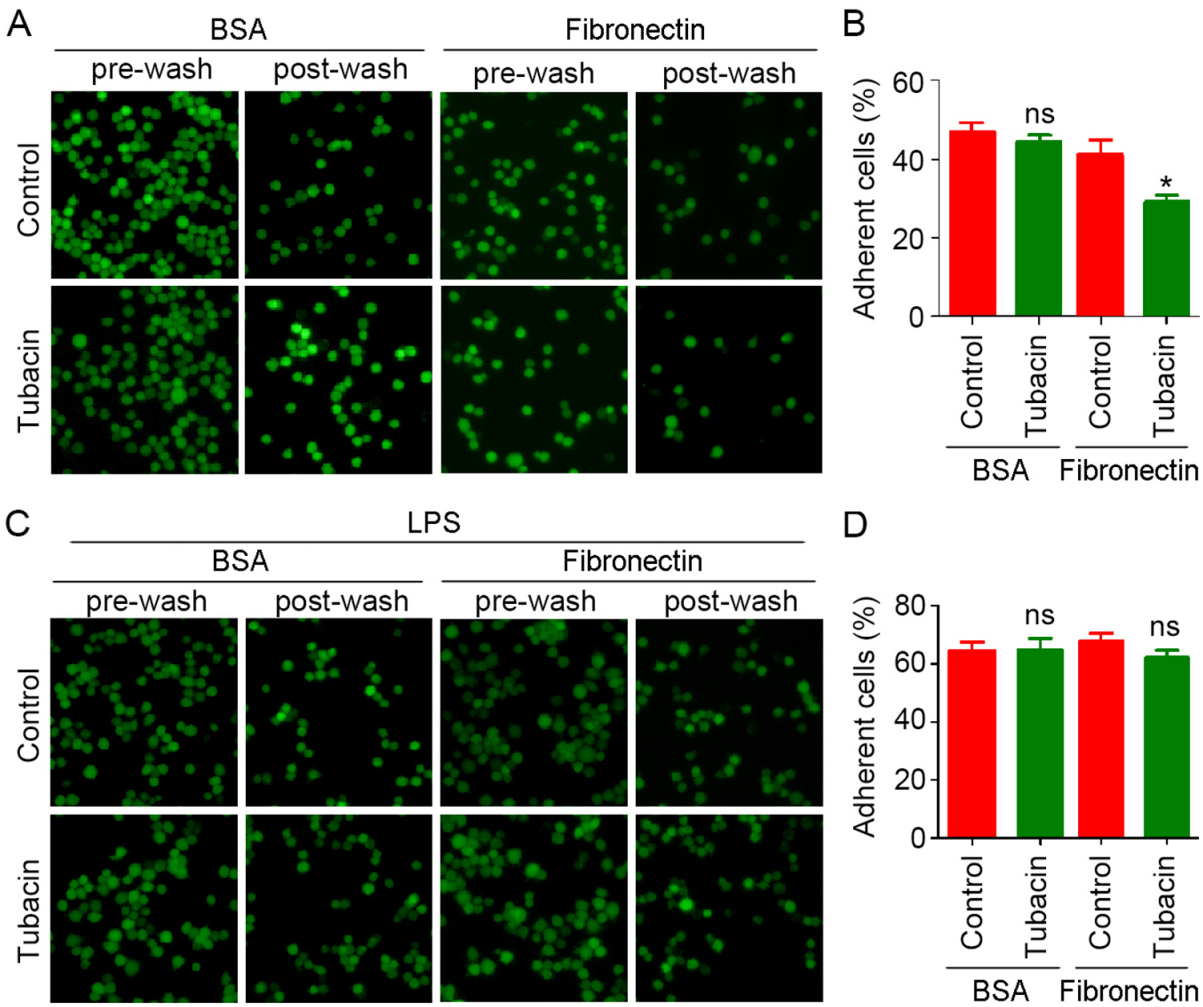
Suppression of HDAC6 activity compromises macrophage adhesion to the extracellular matrix, and this effect is abolished in LPS-stimulated macrophages. (A) RAW264.7 cells labeled with calcein-AM were treated with tubacin and seeded in 96-well plates pre-coated with fibronectin or BSA. Cells were washed twice with PBS, and the fluorescence images of cells were captured by a fluorescence microscope before and after wash. (B) Experiments were performed as in panel A, and the fluorescent intensity was measured. The percentage of adherent cells were quantified by dividing the intensity of post-wash with that of pre-wash. (C, D) Experiments and quantifications were performed as in A and B, except that cells were treated with LPS following tubacin treatment. *, *p*<0.05; ns, not significant (*p*≥0.05).

Activated macrophages infiltrate into the affected sites to remove the pathogens. To investigate how HDAC6 exerts its function in macrophage activation, we examined cell adhesion, one of the most important steps during macrophage infiltration. Cells were seeded onto 96-well plates pre-coated with fibronectin or BSA. As shown in Fig. 4A and 4B, inhibition of HDAC6 with tubacin significantly decreased macrophage adhesion to fibronectin. Furthermore, the effect of HDAC6 on cell adhesion was abolished by LPS stimulation (Fig. 4C and 4D). These data suggest that HDAC6 influences macrophage adhesion to the extracellular matrix in resting macrophages, but not in LPS-activated macrophages.

**Figure 5:**
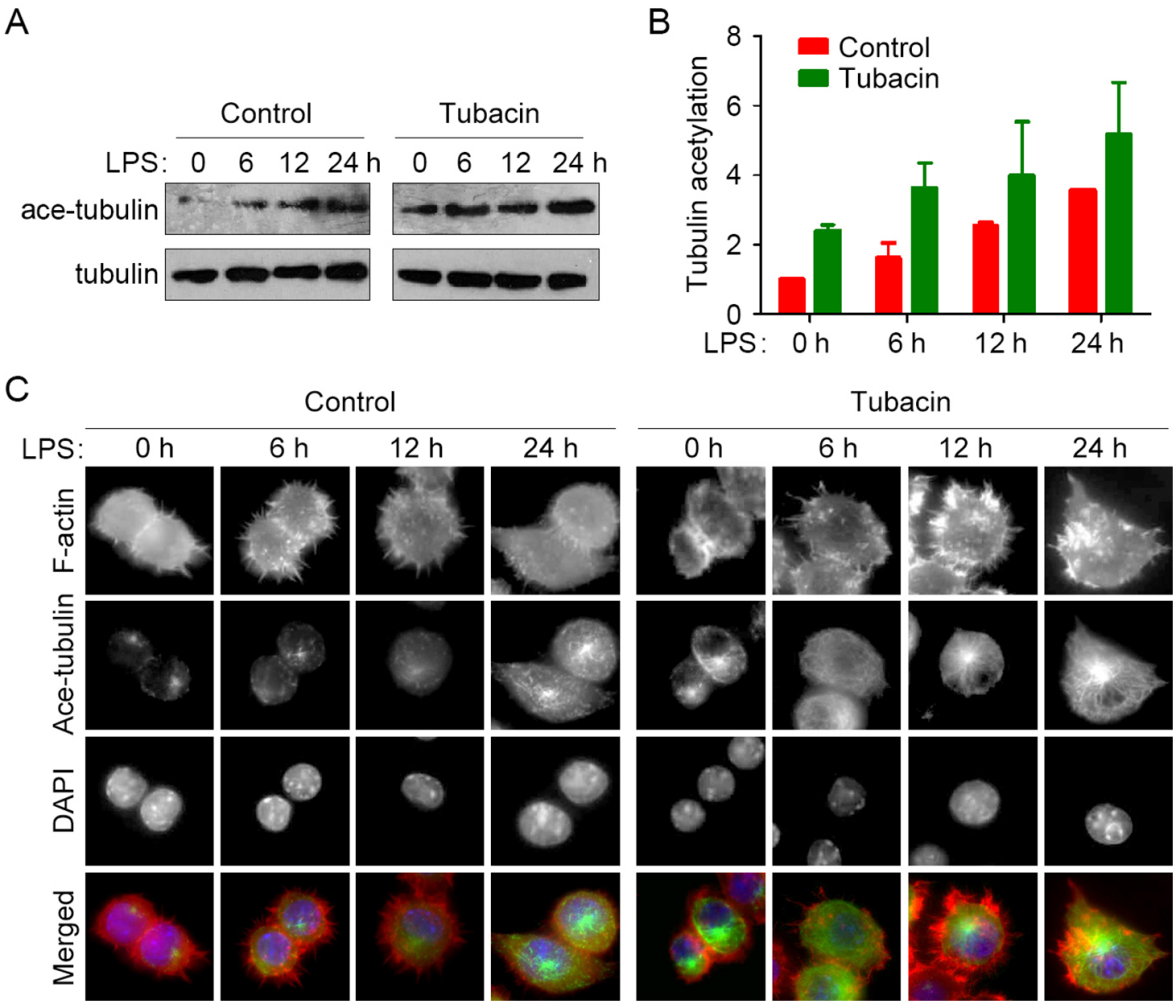
Inhibition of HDAC6 enhances LPS-induced microtubule acetylation during macrophage activation. (A) RAW264.7 cells were treated with tubacin for 4 hours and stimulated with LPS for the indicated time. Acetylated α-tubulin and total α-tubulin were then analyzed by immunoblotting. (B) Experiments were performed as in panel A, and the level of tubulin acetylation was quantified and normalized to the 0-hour LPS stimulation of the control group. (C) RAW264.7 cells were treated with tubacin for 4 hours and stimulated with LPS for the indicated time. F-actin (red), acetylated α-tubulin (green), and nuclei (blue) were then stained, and images were captured with a laser scanning confocal microscope.

We then investigated whether HDAC6 regulates LPS-stimulated macrophage activation by regulating microtubule dynamics. We first examined the level of acetylated microtubules in activated macrophages. As shown in the Fig. 5A and 5B, microtubule acetylation was remarkably elevated upon LPS stimulation, and tubacin significantly enhanced microtubule acetylation compared with the control group. By immunostaining, we found that in the control group, acetylated microtubules mainly concentrated on the microtubule organizing center at 0, 6, and 12 hours of LPS stimulation (Fig. 5C). With the time prolonging to 24 hours, macrophages became activated, accompanied with markedly increased microtubule acetylation (Fig. 5C, control). Consistent with the immunoblotting results, tubacin remarkably elevated microtubule acetylation (Fig. 5C), indicating that microtubule acetylation contributes to the role of HDAC6 in macrophage activation.

## Discussion

Macrophages along with other leukocytes and monocytes, form the first defense line of the innate immune system to prevent the infections of pathogens such as bacteria, viruses, and fungi. In addition to their role in innate immunity, macrophages act as antigen-presenting cells to initiate adaptive immunity [32]. In the physiological conditions, macrophages are maintained in the resting states, whereas upon pathological stimulation macrophages are activated in response to the extracellular cues [14]. Bacterial LPS is recognized by TLR4 of macrophages and induces M1 activation through triggering nuclear factor kappa beta (NF-κB), phosphoinositide 3-kinase (PI3K), and mitogen-activated protein kinase (MAPK) signaling pathways [33], [34]. It is believed that LPS-induced macrophage activation plays a crucial role in many chronic inflammations. In this study, our data show that inhibition of HDAC6 deacetylase activity with the selective inhibitor tubacin or inhibition of HDAC6 expression with specific siRNAs compromises LPS-induced macrophage activation, suggesting that targeting HDAC6 might hold a potential promise for the management of inflammatory diseases.

Activated macrophages exhibit different degrees of elongated morphology compared with the resting cells, with M2 phenotype showing the most elongated cell shape [2]. It is well known that cell shape changes depend on the reorganization of the cytoskeleton [35]–[37], which implicates that macrophage activation towards M1 or M2 may involve cytoskeletal remodeling. In this study, we find that activated macrophages exhibit spreading and elongated cell shape with protrusive structures and this morphology is abolished upon inhibition of HDAC6. Cortactin, an actin scaffolding protein, is localized to active membrane remodeling regions responsible for the formation of filopodia and lamellipodia [38], [39]. Recent studies reveal that HDAC6 regulates actin dynamics and protrusion formation through deacetylation of cortactin [16], [40]. By immunostaining of cortactin and F-actin we show that loss of HDAC6 deacetylase activity does not significantly alter the interaction between F-actin and cortactin. Our further study reveals that HDAC6-mediated macrophage activation is independent of F-actin polymerization and filopodium formation. These findings implicate that HDAC6 probably regulates LPS-induced M1 activation through an actin-independent mechanism.

Activated macrophages migrate to the affected site in response to the pathogens. It is reported that HDAC6 regulates cell migration as a result of its action on cell adhesion [41]. However, it remains elusive whether HDAC6 influences macrophage adhesion and migration. Our data show that inhibition of HDAC6 decreases macrophage adhesion to the extracellular matrix and that this effect is abolished in LPS-stimulated macrophages. We further find that LPS stimulation induces the acetylation of microtubules. Interestingly, suppression of HDAC6 with tubacin also leads to elevated microtubule acetylation. As cytoskeletal reorganization is a dynamic process and is temporally and spatially regulated to accommodate for different cellular behaviors, it is possible that hyperacetylation of microtubules caused by tubacin impairs microtubule dynamics and thereby leads to failure of LPS-induced M1 activation.

To date, HDAC inhibitors have demonstrated great efficacy in several diseases such as cardiovascular disease, cancer, and inflammation [42], [43]. For example, pan-HDAC inhibitors such as TSA and vorinostat have given rise to clinical interest for the treatment of solid tumors [44]. However, due to non-specific inhibition, adverse effect is a major issue for using these drugs in the clinic. In this study, by using an HDAC6 selective inhibitor, we reveal that HDAC6 plays a critical role in LPS-induced macrophage activation. Given that macrophages are important players in many inflammatory diseases, such as atherosclerosis and chronic inflammation, our findings provide a basis for targeting HDAC6 in the management of inflammatory disorders.
